# Novel *Plasmodium vivax* genomes from Brazil identify targets for studies on drug resistance, transmission networks, and parasite evolution

**DOI:** 10.1016/j.lana.2023.100439

**Published:** 2023-01-31

**Authors:** Gustavo Da Silva, Cristian Koepfli

**Affiliations:** University of Notre Dame, Notre Dame, IN, USA

Malaria genomic epidemiology, based on sequencing of whole parasite genomes or typing of specific loci, is a rapidly evolving field of research. Among other applications, researchers and malaria control programs can identify and monitor markers of drug resistance and determine the origin and spread of imported infections. *Plasmodium vivax* is the most widespread human malaria parasite and is predominant outside of Africa. Yet, the number of *P. vivax* genomes sequenced is much lower than for *Plasmodium falciparum*. Only 1072 high-quality *P. vivax* genomes are available in the MalariaGen public database compared to 16,203 for *P. falciparum*.^1^

In this issue of *Lancet Regional Health – America*, Amy Ibrahim and colleagues report *P. vivax* whole genome sequencing data from 51 Brazilian isolates, thus substantially increasing the number of genomes available from Brazil.^2^ More importantly, while previous studies focused mostly on the Amazonian state of Acre, the new genomes were collected in seven Brazilian states, crossing from the Amazon to the Atlantic Forest and representing the variety of transmission settings in the country.

As expected from previous studies, in a global context with a total of 885 samples, Latin American isolates formed their own cluster. Within Latin America, samples from Brazil, Colombia, Guyana, Mexico, Panama, and Peru all formed distinct clusters, with only few exceptions. The study identified 20 SNPs with high power to differentiate isolates from Brazil from other countries. Further validation using genomes not included in the current study will be required to determine whether these SNPs will be useful for the development of genotyping panels to rapidly identify the origin of imported infections.

For the Brazilian malaria control program, determining the origin of infections within the country could be relevant, e.g. to estimate the frequency of parasite importation from the Amazon to other parts of Brazil. Seven clades were identified within Brazil, all except two were predominantly found in the Amazon basin. The study did not attempt to identify SNPs to determine within-country origin, but the genomes could be screened to this aim.

Across the dataset, over 100,000 SNPs were identified. The researchers screened the genomes for regions that showed selection, i.e. they looked for regions where SNPs were frequent or fixed in Brazil or Latin America, but rare in other countries. They found strong selection in genes coding for proteins active in the mosquito. This suggests that parasites underwent selection to adapt to local vector populations as previously shown for *P. falciparum*.^3^ They also found signals of selection in multiple genes involved in reticulocytes invasion. Further research is needed to understand whether this represents adaptations to the local human host population.

Drug resistance is a threat to malaria control, yet given the inability to culture *P. vivax*, genomic markers for resistance are scarce.^4^ The study found SNPs close to fixation in several genes whose homologues in *P. falciparum* affect levels of drug resistance, as well as in additional genes. Many of these signals of selection were found in all Latin American populations, pointing towards parallel evolution in response to the use of the same drugs. These mutations will be prime targets for validation in future treatment efficacy studies. If confirmed to be associated with treatment failure, they will facilitate molecular surveillance of resistance.

Of particular interest are twelve genomes from Sao Paulo that clustered separately from the remaining genomes. The authors speculate that these might be *Plasmodium simium* infections, a malaria parasite found in monkeys as the result of a host switch of *P. vivax* from human to monkey.^5^ *P. simium* outbreaks in humans were reported before in Southeastern Brazil, and it is a matter of debate whether this zoonotic reservoir could impede elimination efforts.^6^ The authors could have tested their hypothesis that they encountered undocumented *P. simium* in humans by comparing their data to published *P. simium* genomes.^7^^,^^8^ Two genes are known to be deleted in *P. simium*. While sequencing coverage was too poor to check for their absence; this could have been tested through qPCR.

Lastly, the data might help to clarify the origins of malaria in the Americas. It is unclear whether malaria was present before the arrival of Europeans following Columbus and possibly sailors from the western Pacific. The mitochondrial genome of a now-extinct Spanish *P. vivax* population resembles closely to present-day South American parasites.^9^ Likewise, Polynesians might have reached South America in pre-Columbian times and could have brought *P. vivax* with them.^10^ Ibrahim and colleagues identified four major clusters of *P. vivax* in South America, and two in Brazil. Further research will be needed to show whether they represent different routes of introduction.

In summary, the rich dataset presented by Ibrahim and colleagues lays the foundation for future studies on drug resistance, routes of transmission, and more generally the evolutionary forces that shape *P. vivax* populations.

## Contributors

GDS and CK contributed to writing this commentary.

## Declaration of interests

The authors confirm that no conflicts of interest exist.
